# Surge in Anaplasmosis Cases in Maine, USA, 2013–2017

**DOI:** 10.3201/eid2602.190529

**Published:** 2020-02

**Authors:** Susan P. Elias, Jessica Bonthius, Sara Robinson, Rebecca M. Robich, Charles B. Lubelczyk, Robert P. Smith

**Affiliations:** Maine Medical Center Research Institute, Scarborough, Maine, USA (S.P. Elias, R.M. Robich, C.B. Lubelczyk, R.P. Smith, Jr.);; University of Southern Maine, Portland, Maine, USA (J. Bonthius);; Maine Center for Disease Control and Prevention, Augusta, Maine, USA (S. Robinson)

**Keywords:** *Anaplasma phagocytophilum*, anaplasmosis, deer tick, *Ixodes scapularis*, Maine, tickborne disease testing, bacteria, vector-borne infections, zoonoses

## Abstract

Incidence of human granulocytic anaplasmosis is rising in Maine, USA. This increase may be explained in part by adoption of tick panels as a frequent diagnostic test in persons with febrile illness and in part by range expansion of *Ixodes scapularis* ticks and zoonotic amplification of *Anaplasma phagocytophilum.*

Lyme disease is the most common vectorborne disease in Maine. *Borrelia burgdorferi*, the agent of Lyme disease, is transmitted through the bite of infected blacklegged ticks (*Ixodes scapularis*). Lyme disease cases in Maine increased from a single case in 1986 to 1,844 in 2017, reflecting the northward range expansion of *I. scapularis* ticks (*1*). *Anaplasma phagocytophilum,* the cause of human granulocytic anaplasmosis (HGA), is also transmitted by *I. scapularis* ticks and is the second most common tickborne illness in Maine. Only 45 HGA cases were reported during 2000–2008 (*2*), but case reports rose dramatically during 2013–2017, generating media attention (*3**,**4*). The Maine Center for Disease Control and Prevention (MECDC) reported 663 cases of anaplasmosis in 2017, a 605% increase from 94 cases in 2013, in contrast with Lyme disease cases, which increased by only 33% (1,384 in 2013 to 1,844 in 2017) (*5*).

We sought to determine whether the increase in anaplasmosis cases reflected broader geographic transmission of *A. phagocytophilum* from ticks to humans through range expansion of *I. scapularis* ticks, increased testing effort through increased use of tick panels that detect multiple pathogens by PCR, or both. Evidence for increased transmission would include geographic range expansion of HGA incidence and hospitalizations. Evidence of increased testing effort would be increased use of tick panels, which could lead to discovery of mild *A. phagocytophilum* infections, especially pediatric cases, because HGA in children is generally a mild illness (*6*).

## The Study

MECDC provided the number of confirmed and probable cases for Maine residents during 2008–2017, available by county of residence and age of onset. For 2013–2017, we obtained the annual number of hospitalizations for Lyme disease and HGA for Maine residents, with age and county of residence at admission, from the Maine Health Data Organization.

We obtained the annual number of multipathogen (including HGA) PCR tick panel orders during 2013–2017 from NorDx and Mayo Medical Laboratories (MML). NorDx (Scarborough, ME, USA) started using its panel for the agents of HGA and babesiosis in 2015 (H. Webber, NorDx, pers. comm., 2018 Sep 12). All orders were for Maine patients (travel history not specified). MML provided data from 2 branch laboratories, Mayo Clinic Rochester (MCR; Rochester, MN, USA) and Mayo Medical Laboratories, New England (MMLNE; Andover, MA, USA). MML has offered the panel for ≈10 years; the panel contains a PCR test for the agents of human monocytic ehrlichiosis, HGA, babesiosis, and *Borrelia miyamotoi* infections (B. Pritt, MML, pers. comm., 2018 Aug 3). MML data comprised specimens sent to MML from Maine clients, without patient residence or travel history (B. Pritt, A. Boerger, MML, pers. comm., 2018 Oct 8). We were unable to obtain data from other laboratories; however, MML and NorDx combined accounted for 72% of HGA test reports sent to MECDC during 2013–2017. The MML panel had sensitivity and specificity of 1 for detection of *A. phagocytophilum* compared with standard PCR (*7*). The NorDx panel had sensitivity and specificity of 1 compared with panels of MML and other laboratories (H. Webber, pers. comm., 2019 Aug 29).

The HGA incidence rate, hospitalization rate, complications, and death rate increase with age (*8*), whereas Lyme incidence has a bimodal distribution, with peaks in young children and older adults (*9*). We tabulated HGA cases and incidence for 2008–2017 overall and by age class and tabulated HGA hospitalizations 2013–2017 overall and by age class and annual laboratory testing effort. For comparison, we included annual overall Lyme incidence and hospitalizations. We compared percentage changes from 2013 to 2017 in disease incidence and hospitalizations. To visualize geographic expansion of HGA, we plotted side-by-side maps of county-level incidence and population-adjusted hospitalization rates for 2013 versus 2017.

During 2013–2017, a total of 1,505 anaplasmosis cases were reported (*10*). Of these, 85.6% (1,289) were confirmed (1,286 by PCR and 3 by 4-fold antibody titer increase) and 14.4% (216) probable (203 with a single titer result, 8 with <4-fold titer increase, 5 with morulae visualization). Statewide, anaplasmosis incidence rose from 7 cases/100,000 persons in 2013 to 50 cases/100,000 persons in 2017, a 602% increase, compared with a 33% increase for Lyme disease incidence (Table 1). Hospitalizations for HGA rose from 36 in 2013 to 119 in 2017, a 231% increase, compared with a 27% decline in hospitalizations for Lyme disease (Table 2). Combined tick panel use by MML and NorDx rose from 773 in 2013 to 9,157 in 2017, a 1,085% increase (Table 2). 

**Table 1:** Number of HGA and Lyme disease cases and incidence, Maine, USA, 2008–2017*

Year	HGA											
	Cases					Incidence					Lyme	
	All ages	0–17 y	18–64 y	>65 y		All ages	0–17 y	18–64 y	>65 y		Cases	Incidence†
2008	17	0	11	6		1.3	0	1.3	3.0		909	68.3
2009	15	0	9	6		1.1	0	1.1	2.9		976	73.4
2010	17	1	10	6		1.3	0.4	1.2	2.8		752	56.6
2011	26	0	12	14		2.0	0	1.4	6.5		1013	76.3
2012	52	0	37	15		3.9	0	4.4	6.6		1113	83.7
2013	94	0	56	38		7.1	0	6.7	16.2		1384	104.2
2014	191	10	102	79		14.4	3.9	12.3	32.5		1411	106.1
2015	185	9	92	84		13.9	3.5	11.2	33.6		1215	91.4
2016	372	9	206	157		27.9	3.5	25.2	60.8		1497	112.4
2017	663	13	304	346		49.7	5.1	37.3	129.6		1844	138.5
Change 2013–2017‡	605%		443%	811%		602%		454%	701%		33%	33%

*Case data provided by the Maine Center for Disease Control and Prevention. HGA, human granulocytic anaplasmosis. †Cases/100,000 population.  ‡Percentage increase 2013–2017 was calculated when there were nonzero data for 2013.

**Table 2:** Number of hospitalizations for human granulocytic anaplasmosis (HGA) and Lyme disease, and number of PCR-based tickborne disease panels, 2013–2017*

Year	Hospitalizations							Tick panels†				
	HGA					Lyme, all ages						
	All ages	0–17 y	18–64 y	>65 y				MML-R	MML-NE	Total MML	NorDx	All
2013	36	0	9	27		66		0	773	773	0	773
2014	75	0	25	50		55		0	1,479	1,479	0	1,479
2015	68	0	19	49		45		0	1,066	1,066	875	1,941
2016	123	0	42	81		47		596	122	718	5,259	5,977
2017	119	0	24	95		48		973	0	973	8,184	9,157
Change 2013–2017	+231%		+167%	+252%		−27%						+1,085%

*Hospitalizations are Maine hospital inpatient encounters, provided through the Maine Health Data Organization. HGA, human granulocytic anaplasmosis; MML, Mayo Medical Laboratories. †Tick panels performed at Mayo Clinic Rochester, Rochester, MN, USA (MML-R); MML, New England, Andover, MA, USA (MML-NE, closed in 2016); and NorDx, Scarborough, ME, USA.

Among 39 pediatric HGA cases, 1 occurred in 2010 and the remaining 38 during 2014–2017, representing 1.7%–5.2% of total cases per year during 2014–2017 (Table 1). Even though hospitalizations increased for persons 18–64 and >65, there were no hospitalizations for children.

**Figure 1:**
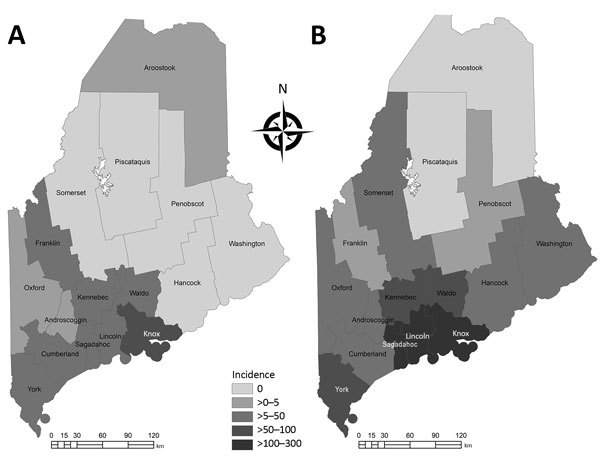
Human granulocytic anaplasmosis incidence (cases/100,000 persons), Maine, USA, 2013 (A) and 2017 (B). Statewide incidence increased 602% during 2013–2017.

**Figure 2:**
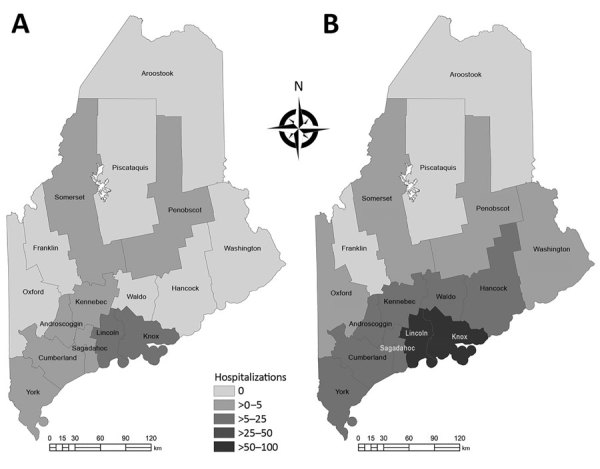
Hospitalizations (per 100,000 persons) for human granulocytic anaplasmosis, Maine, USA, 2013 (A) and 2017 (B). Statewide hospitalizations increased 231% during 2013–2017.

Anaplasmosis incidence and hospitalizations underwent geographic range expansion during 2013– 2017 (Figures 1, 2). Anaplasmosis incidence was highest in Lincoln and Knox Counties, in Maine’s midcoast region, where incidence ranged from 29 cases/100,000 persons in 2013 to 278 cases/100,000 persons in 2017 (Figure 1, panels A, B).

## Conclusions

We conclude that the surge in anaplasmosis incidence in Maine, an increase of 602% from 2013 to 2017, was a combination of increased transmission and testing effort, although we cannot partition the relative contribution of each. The 231% rise in hospitalized patients with HGA and the geographic expansion of HGA incidence and hospitalization indicate increased transmission. Range expansion of *I. scapularis* ticks in Maine likely has contributed to the rise in HGA cases in areas where this tick species is emergent (i.e., a recent colonizer). In addition, zoonotic amplification of *A. phagocytophilum* is likely occurring where *I. scapularis* ticks are established. Because of less efficient enzootic transmission, human infection with *Babesia microti*, the agent of babesiosis, lags behind *B. burgdorferi* transmission over time and space (*11*). Less efficient enzootic transmission of *A. phagocytophilum* also may be the case, but we know of no confirmatory studies.

Concurrent to increased transmission was the 1,085% increase in tickborne disease panel testing performed by the 2 major providers of testing results to Maine during 2013–2017. Increased testing effort may reflect increased clinician and patient awareness and ready availability of tickborne disease panels that detect multiple pathogens. These panels may lead to detection of mild *A. phagocytophilum* infections or co-infections in persons with nonspecific febrile illness, as suggested by increased detection of less severely ill persons, such as children. Thirty-eight of 39 pediatric HGA cases were reported after 2013, but there were no pediatric hospitalizations. Before the use of panels, pediatric HGA cases may have been ascribed to another illness with similar symptoms.

Studies relying on diagnostic tests are subject to test sensitivity and specificity. PCR is the most effective diagnostic test during early-stage *A. phagocytophilum* infection with high sensitivity and specificity (*12**,**13*). In this study, false positive PCR results were unlikely, based on test specificities reported by Mayo and NorDx. 

Collaboration among all state health departments and testing laboratories across New England could help extend our findings. Vermont cases increased 1,078%, from 37 in 2013 to 399 in 2017 (*14*), and New Hampshire cases increased 260%, from 88 in 2013 to 317 in 2017 (*15*). Correlation between incidence and testing effort at the county level would corroborate a relationship between rising tickborne diseases and testing effort, if panel data included patient county of residence and travel history. Corroborating datasets on density of *A. phagocytophilum*–infected *I. scapularis* ticks would also help clarify the risks posed to human health.
